# From physiology to salt marsh management challenges with sea level rise: the case of native *Spartina foliosa*, invasive *S. densiflora* and their hybrid

**DOI:** 10.1093/conphys/coaa053

**Published:** 2020-07-01

**Authors:** Blanca Gallego-Tévar, Procopio Peinado-Torrubia, Rosario Álvarez, Brenda J Grewell, Jesús M Castillo

**Affiliations:** 1 Departamento de Biología Vegetal y Ecología, Universidad de Sevilla, Ap 1095, 41080 Sevilla, Spain; 2 USDA-ARS, Invasive Species and Pollinator Health Research Unit, Department of Plant Sciences MS-4, 1 Shields Avenue, University of California, Davis, CA 95616, USA

**Keywords:** environmental stress, hybrid, inundation, PEPC, polyploid, salinity, transgressive traits

## Abstract

Sea level rise (SLR) imposes increasing salinity and inundation stresses in salt marshes which simultaneously face invasions by exotic plant species. We aimed to improve and apply knowledge on the ecophysiological responses of halophytes to SLR to conservation management of salt marshes. In a mesocosm experiment, we measured and compared phosphoenolpyruvate carboxylase (PEPC) activity and related functional traits of the California-native *Spartina foliosa*, invasive *S. densiflora* and their hybrid *S. densiflora × foliosa* in response to increasing levels of salinity and inundation. *S. foliosa* was moderately sensitive to salinity, showing a 57% reduction in PEPC specific activity from freshwater to hypersalinity. This native species compensated for the reduction of PEPC activity with increased salinity through 80% higher enzyme activation by phosphorylation. PEPC functional trait responses of *S. foliosa* were mostly independent of inundation depth. In view of these results, managers should conserve undeveloped lands for accommodation space above current high tide lines to facilitate colonization of stress-tolerant *S. foliosa*. Our results on functional responses of PEPC traits recorded high sensitivity to salinity for *S. densiflora*. This was reflected by 65% lower PEPC specific activity together with increasing accumulation of free proline (+96%) and total proteins (+23%) with elevated salinity. These results suggest prioritized eradication of *S. densiflora* populations in brackish habitats. Measured PEPC responses support the high stress tolerance of the *S. densiflora* × *foliosa* hybrid. PEPC traits for the hybrid were mostly independent of salinity and inundation. The hybrid showed higher PEPC-specific activity than *S. foliosa* (+70%) and *S. densiflora* (+15%) in freshwater under intermediate inundation. Results suggest that eradication of the hybrid should be the highest management priority. Our study shows that the responses of key functional physiological traits to environmental stresses serve as biological indicators that can guide ecosystem management practices in a scenario of climate change.

## Introduction

Sea level rise (SLR) due to global warming is increasing salinity, and inundation depth and duration in the world’s salt marshes (IPCC, 2015). These environmental changes are highly significant since salinity and flooding are among the main abiotic stress factors determining the performance and distribution of halophytes in salt marshes (Engels and Jensen, 2010). In this context, halophytes may respond to SLR by migrating to less stressful habitats or survive *in situ* through genetic adaptation to changing conditions and phenotypic plasticity (Xue *et al*., 2018). In addition to SLR, salt marshes are being impacted by other human-mediated processes such as biological invasions. Global climate change and invasive species can also have interacting effects that compound uncertainty associated with each individual stress driver (Hellman *et al*., 2008). Exotic invasive species displace local biodiversity in salt marshes (Gedan *et al*., 2009). Sometimes, alien species hybridize with native species producing hybrids with high stress tolerance and competitive ability that also displace native biodiversity in salt marshes (Wong *et al*., 2018; Williams *et al*., 2019). This improved performance of hybrids may be related to transgressive traits due to non-additive gene expression (Favre and Karrenberg, 2011). Interacting environmental changes can decrease the effectiveness of invasive plant management, and it is important for conservation managers to identify which invasive species are likely to change (Hellman *et al*., 2008). Therefore, improved knowledge of functional and evolutionary traits supporting the invasiveness and relative impacts of exotic plant species is a priority (Drenovsky *et al*., 2012).

In the context of ongoing global environmental changes, preservation of salt marshes is essential since they carry out many important ecosystem services such as mitigating climate change by sequestrating atmospheric carbon, providing food, regulating water and air quality, buffering the impacts of storms and tsunamis and offering natural spaces for recreational activities (Moomaw *et al*., 2018). Choosing and prioritizing salt marsh conservation goals is not an easy task, but knowledge of ecophysiological mechanisms underlying the survival and persistence of key halophyte species can inform decision-making to preserve ecosystem functions. In this context, improved knowledge of ecophysiological responses of both native and invasive species to a changing environment can provide scientifically based insight for design, prioritization and implementation of conservation management practices (French *et al*., 2017).

*Spartina* species (cordgrasses; Poaceae) are among the most widely distributed and abundant halophytes in salt marshes around the world. Many *Spartina* taxa behave as invasive alien species in their non-native geographical ranges where they have hybridized with resident native congeners forming transgressive hybrids (Strong and Ayres, 2013). However, some F1 *Spartina* hybrids are initially sterile, but research suggests they may become fertile and very competitive allopolyploid species (Aïnouche *et al*. 2004). Cordgrasses have a C4 photosynthetic metabolism, with phosphoenolpyruvate carboxylase (PEPC) playing a key role in CO_2_ assimilation and other metabolic pathways. PEPC is regulated by allosteric positive (e.g. glucose-6-phosphate) and negative (e.g. L-malate) effectors (Jiao *et al*., 1991). In addition, PEPC enzymatic activity is regulated by reversible protein phosphorylation at its N-terminal domain and this process is controlled by a highly regulated Ca^2+^-independent protein-Ser/Thr kinase (Jiao *et al*., 1991). Light activates the kinase and consequently causes an increase in the activity and a decrease in the L-malate sensibility of PEPC. The low sensibility to L-malate indicated a dephosphorylated PEPC at freshwater conditions (Jiao and Chollet, 1992; Wang and Chollet, 1993). This regulatory phosphorylation depends not only on light but also on several abiotic factors such as salinity, carbon dioxide or inundation levels (Li and Chollet, 1994; Echevarría *et al*., 2001; Yordanova and Popova, 2007; Mateos-Naranjo *et al*., 2010). Thus, environmental changes in salt marshes due to SLR may alter PEPC activity in C4-halophytes and greatly influence changes in carbon fixation and ultimately change plant growth. In this sense, a recent study analyzed the effects of salinity on functional traits of PEPC in the European native *Spartina maritima* (Curtis) Fernald and the South American invasive *Spartina densiflora* Brongn. and their reciprocal hybrids and documented the development of some transgressive traits by the hybrids (Gallego-Tévar *et al*., 2019a). Studies of other *Spartina* hybrids (i.e. sterile *S. x townsendii*, a hybrid between *Spartina alterniflora* Loisel. and *S. maritima* in England; fertile *S. alterniflora x foliosa* hybrid in the San Francisco Estuary) have also revealed transgressive traits (Strong & Ayres, 2013). We reviewed 28 studies related to the response of *S. foliosa*, *S. densiflora* and their hybrids to salinity, 20 works related to inundation, and only one publication analyzing the combined effect of salinity and inundation on these taxa (Supplementary information: Literature review). To our knowledge, no previous studies have analyzed the combined effects of both salinity and inundation on PEPC performance and halophyte response.

Our study system included the California-native *Spartina foliosa* Trin. colonizing low to middle intertidal marshes, and invasive *S. densiflora* and their sterile F1 hybrid *S. densiflora x foliosa* growing mostly in middle/high marshes in the San Francisco Estuary (CA, USA) (Gallego-Tévar *et al*., 2020). Recent publications analyzing functional responses to salinity, inundation and their interaction have characterized *S. foliosa* as a stress-tolerant species, *S. densiflora* as a fast-growing species able to take advantage of low to moderate abiotic stress conditions and their invasive cross as a transgressive hybrid with high stress tolerance (Gallego-Tévar *et al*., 2019b, 2020). Our primary goals were to (i) improve present knowledge on the ecophysiological responses of these three halophytes to SLR and (ii) apply this new biological information to the management of salt marshes in the context of environmental change. We performed a mesocosm experiment where, for the first time, PEPC activity and its regulation were recorded in response to the combined stress effects of salinity and inundation depth mimicking a SLR scenario. In addition, foliar free proline and malondialdehyde (MDA) concentrations were recorded as biological indicators of salt stress and oxidative damages, respectively (Mulholland & Otte, 2001; De Azevedo Neto *et al*. 2006). We hypothesized that native *S. foliosa* would have higher tolerances to salinity and flooding than invasive *S. densiflora*, whereas their hybrid would have greater tolerance than both parental species to both abiotic stresses due to influences of PEPC enzymatic levels. Our experimental results, in combination with the literature review (Supplementary information: Literature review), were then used to make conservation recommendations for native *S. foliosa* and to predict and prioritize counter measures in response to the invasion of *S. densiflora* and its hybrid.

## Material and methods

### Studied taxa and plant material

*Spartina foliosa* (2n = 62 chromosomes; Ayres *et al*., 2008) is the only native cordgrass in estuaries along the North American Pacific Coasts of California (USA) and Baja California (Mexico) (Mobberly 1956; Ayres *et al.*, 2003). It plays a key role in primary succession of salt marshes and provides ecosystem services such as improved water quality, sediment accretion and habitat functions for fishes and wading birds (Ayres *et al*., 2003) (see Supplementary Fig. 1). In the San Francisco Estuary, *S. foliosa* has hybridized with invasive *S. alterniflora* and with invasive *S. densiflora* (Daehler and Strong, 1997; Ayres *et al*., 2008). For our experiment, we obtained *S. foliosa* individuals from middle to low marshes in the Carquinez Strait, in the northern reach of the San Francisco Estuary (38°57′57″ N, 122°11′36″ W).

*Spartina densiflora* (2n = 70) is a South American cordgrass that has invaded salt marshes in the Southwest Iberian Peninsula and along the Pacific Coast of North America (Bortolus, 2006) from the San Francisco Estuary north to British Columbia (Castillo *et al*., 2014). Invasive populations of *S. densiflora* show high phenotypic plasticity and low genetic diversity along the Pacific Coast of North America, where in places, the species co-occurs with *S. foliosa* (Castillo *et al*., 2014, 2016, 2018; Grewell *et al*., 2016). The sterile hybrid *Spartina densiflora* × *foliosa* (diploid; 2n = 65 chromosomes) is primarily found in an overlapping range with *S. densiflora* within middle elevation salt marshes in the San Francisco Estuary (Ayres *et al.*, 2008) (see Supplementary Fig. S1). This hybrid may have higher salinity tolerance than both parental species (Lee *et al*., 2016). *S. densiflora* and the hybrid *S. densiflora × foliosa* were collected from middle intertidal marshes in the Corte Madera Creek tributary to the San Francisco Estuary (37°56′27″ N, 122°31′2″ W).

### Experimental design

*Spartina* rhizomes were cleaned and classified into weight classes to obtain similar-size experimental individuals according to the growth form of the different taxa (80–120 g for *S. foliosa* rhizomes; 230–280 g for *S. densiflora*; 20–50 g for the hybrid) at the Aquatic Weed Research Facility, University of California, Davis, in March 2017. Clean and standardized rhizomes were transplanted to 3.1 litre pots (pot size: 15 cm diameter × 17.5 cm height) containing sterile sand. Pots were sub-irrigated with freshwater for 2 months prior to arrangement in 16 500-l plastic mesocosms (1.3 m × 0.8 m × 0.6 m) (Rubbermaid, Atlanta, GA) for exposure to different salinity and inundation treatments. Treatments were randomly assigned within a randomized complete block design with treatments (4 salinity levels × 3 inundation levels × 3 taxa) nested within the 16 mesocosms. The split-plot, full-factorial experimental design included salinity as the whole plot factor, with each salinity level replicated 4 times. Inundation level was randomly assigned within salinity mesocosms as the subplot (within plot) factor, and the three focal taxa were nested within the subplots (*n* = 4 plants per taxon and treatment combinations). Salinity treatments ranged from freshwater to hypersalinity (0.5, 10, 20 and 40 ppt); treatments were prepared using a 20% Hoagland’s nutrient solution and Hoagland’s solution plus sea salts (Instant Ocean®, Aquarium Systems Inc., Mentor, OH) to achieve desired concentrations. EcoPond Clear biological product (Grow More Inc., Gardena, CA) was added to reduce algal proliferation. Salinity was gradually increased by 10 ppt per week to avoid osmotic shock in the higher salinity treatments. Three permanent inundation treatments were established: deep inundation (55.0 cm deep; pots placed on bottom of tank), intermediate inundation (35.5 cm deep; pots placed on concrete stands within tanks) and shallow inundation (4.5 cm deep; pots placed on stacked concrete stands within tanks). The experiment was carried out for 31 days (8–June 8 May 2017) in a glasshouse with controlled air temperature between 21–25°C. The natural photoperiod was extended to 12-h daily using high-intensity discharge lights (GE Lucalox LU1000/ECO HPS 1000 W, PARsource, Petaluma, CA). The photon flux density measured by a photometer (LI-COR LI-250A light meter; LI-COR Inc., Lincoln, NE) was 500 μmol m^−2^ s^−1^ at the canopy level and 100 μmol m^−2^ s^−1^ at the bottom of the mesocosms at midday. Plant material was collected at midday from the mid-section of randomly chosen flag leaves (first unfolded adult leaf from the apical leaf; ca. 2 g per plant) and lyophilized (*n* = 3–4 per treatment combination).

### Abiotic stress indicators: proline and MDA quantification

Free proline is a compatible organic solute that is a measure of plant response to salt stress, as reported previously for *Spartina* species (Mulholland & Otte, 2001). Foliar free proline content was determined following Bates *et al.* (1973). Lyophilized leaves (0.5 g) were homogenized in 10 ml of 3% sulfosalicylic acid and centrifuged at 15 000g for 5 min. Supernatant (2 ml) was combined with glacial acetic (2 ml) acid and acid-ninhydrin (2 ml) and boiled at 100°C for 1 h. Reaction was stopped in ice, and toluene (2 ml) was added. The upper toluene phase was analysed spectrophotometrically at 517 nm. Free proline concentration was calculated from a standard curve of L-proline.

Foliar MDA concentration was recorded to evaluate oxidative damage in relation to lipid peroxidation accumulation as a response to salinity and inundation stress (De Azevedo Neto *et al*. 2006; Zhu *et al*., 2019). MDA was assayed following Buege and Aust (1978). Lyophilized leaves (0.1 g) were homogenized in TCA–TBA reagent (2 ml; 20% (w/v) trichloroacetic acid (TCA) and 0.5% (w/v) 2-thiobarbituric acid (TBA)) and centrifuged at 13 800g for 2 min. The supernatant was boiled at 90°C for 30 min, then cooled and centrifuged at 15 000 g for 15 min. The absorbance of the supernatant was measured at 532 nm for MDA and at 600 nm for the non-specific absorption. MDA concentration was calculated using its molar extinction coefficient (*ε* = 155 mM^−1^ cm^−1^).

### PEPC activity, L-malate test and soluble protein quantification

Lyophilized leaf tissue (0.2 g) was ground with 1 ml of extraction buffer containing 0.1 M Tris–HCl pH 7.5, 20% (v/v) glycerol, 1 mM EDTA, 10 mM MgCl_2_ and 14 mM mercaptoethanol. The homogenate was centrifuged at 17 000 g for 2 min, and the supernatant was used immediately as a clarified protein extract to determine the PEPC activity and sensitivity of PEPC to L-malate. PEPC activity was measured spectrophotometrically at optimal and suboptimal pH (8.0 and 7.3, respectively) using the NAD-malate dehydrogenase coupled assay containing 2.5 mM phosphoenolpyruvate (PEP), 1 mM NaHCO_3_^−^, 5 mM MgCl_2_, 5 units of NAD-malate dehydrogenase, 0.2 mM NADH and 100 mM Hepes/KOH (Echevarria *et al.*, 1994). An enzyme unit (U) was defined as the amount of PEPC that catalyzes β-carboxylation of 1 μmol of PEP min^−1^ at pH 8 and 30°C. Malate sensitivity was determined at suboptimal pH 7.3 in the presence of various concentrations of L-malate, where the malate inhibition of PEPC activity was expressed as IC_50_ (50% inhibition of initial PEPC activity by L-malate). Previous studies validated the L-malate test as reflecting PEPC phosphorylation state (Feria *et al*., 2008). A high IC_50_ is related to a high degree of PEPC phosphorylation (Echevarria *et al*., 1994). Protease and phosphatase inhibitors were not added to the extraction buffer to avoid PEPC activity lost since PEPC activity in leaf extracts was measured rapidly and very diluted (5 μl of crude extract in a final volume of 1000 μl); similar results have been obtained with and without adding inhibitors (Echevarría *et al*., 1990; Gallego-Tévar *et al*., 2019a). Extracts were not desalted as previous studies have shown similar results with and without desalting (Rodríguez-Penagos and Muñoz-Clares, 1999; Gandullo *et al*., 2019; Gallego-Tévar *et al*., 2019a). The total protein amount was determined following the colorimetric method of Bradford (1976), using bovine serum albumin as standard. PEPC activity was expressed as units per gram of protein (apparent specific activity).

### Statistical analysis

Analyses were carried out using Sigma-Plot (Systat Software Inc., Point Richmond, CA; Windows version 12.0). Results were considered significant when *P* ≤ 0.05. Kolmogorov–Smirnov and Levene tests were used to verify the normality and homogeneity of variance of the data series. Data series for PEPC apparent specific activity and total protein amount were transformed using the function √*x* to reach normality and homogeneity of variance. Each variable assayed was analysed using three-way analysis of variance (ANOVA) with taxa, salinity and inundation treatments as grouping factors. Tukey’s honesty significant different (Tukey–HSD) test was used as *post hoc* analysis. Relationships between PEPC traits, total protein content and proline and MDA concentrations among them and with inundation and salinity treatments were studied for every taxon using the Pearson correlation coefficient (*r*).

## Results

### Abiotic stress indicators: proline and MDA accumulation

**Figure 1:**
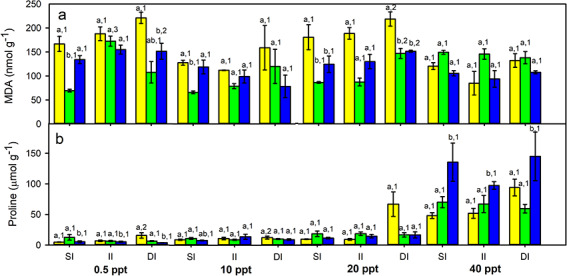
Foliar free proline (**a**) and malondialdehyde (MDA) (**b**) concentration for native *Spartina foliosa* (yellow columns), invasive *S. densiflora* (blue columns) and their hybrid *S. densiflora × foliosa* (green columns) exposed to four salinity treatments (0.5, 10, 20 and 40 ppt) at three inundation depths (shallow inundation (SI), 4.5 cm deep; intermediate inundation (II), 35.5 cm deep; deep inundation (DI), 55.0 cm deep). Values are mean ± SEM (*n* = 4 for proline and 3 for MDA). Different letters indicate significant differences among taxa for the same inundation treatment at every salinity. Different numbers show significant differences among inundation treatments at the same salinity for each taxon (three-way ANOVA, Tukey–HSD, *P* < 0.05)

Foliar free proline concentration showed significant differences among salinity and inundation treatments, and for taxa × salinity and taxa × inundation interactions (Table 1). Proline accumulation increased markedly with salinity for every taxon (Table 2). Proline concentration decreased with increasing total protein content for *S. foliosa* and the hybrid, showing the opposite response measured for *S. densiflora*. *S. densiflora* accumulated more proline than the other two taxa at hypersalinity under every inundation treatment. In contrast, *S. foliosa* and the hybrid increased their proline concentration at deeper inundations, especially at 20 and 40 ppt salinity (Fig. 1a; Table 2).

**Table 1:** Three-way ANOVA for plant traits of native *Spartina foliosa*, invasive *S. densiflora* and their hybrid *S. densiflora × foliosa* with taxa, salinity, inundation depth as grouping factors

	**Taxa**	**Salinity**	**Inundation**	**Taxa × salinity**	**Taxa × inundation**	**Salinity × inundation**	**Taxa × salinity × inundation**
**Free proline (μmol gDW** ^**−1**^ **)** (*n* = 4)	*F*_2,108_ = 0.23, *P* > 0.05	***F***_**3,108**_ **= 264.59, *P* < 0.001**	***F***_**2,108**_ **= 6.54, *P* < 0.005**	***F***_**6,108**_ **= 5.86, *P* < 0.001**	***F***_**4,108**_ **= 10.34, *P* < 0.001**	*F*_6,108_ = 1.75, *P* > 0.05	*F*_12,108_ = 1.71, *P* > 0.05
**Malondialdehyde (nmol gDW** ^**−1**^ **)** (*n* = 3)	***F***_**2,71**_ **= 21.54, *P* < 0.001**	***F***_**3,71**_ **= 16.50, *P* < 0.001**	***F***_**2,71**_ **= 6.65, *P* < 0.005**	***F***_**6,7 1**_ **= 9.64, *P* < 0.001**	*F*_4,71_ = 2.16, *P* > 0.05	***F***_**6,71**_ **= 3.98, *P* < 0.005**	*F*_12,71_ = 1.60, *P* > 0.05
**PEPC apparent specific activity (μmol PEP min** ^**−1**^ **mg**^**−1**^**)** (*n* = 3–4)	***F***_**2,73**_ **= 59.77, *P* < 0.001**	***F***_**3,73**_ **= 51.95, *P* < 0.001**	*F*_2,73_ = 0.03, *P* > 0.05	***F***_**6,73**_ **= 7.34, *P* < 0.001**	*F*_4,73_ = 2.16, *P* > 0.05	***F***_**2,73**_ **= 1.75, *P* < 0.001**	***F***_**12,73**_ **= 3.93, *P* < 0.001**
**Level PEPC activation by phosphorylation (IC**_**50**_ **; mM)** (*n* = 3–4)	***F***_**2,78**_ **= 22.83, *P* < 0.001**	***F***_**3,78**_ **= 15.65, *P* < 0.001**	*F*_2,78_ = 1.65, *P* > 0.05	***F***_**6,78**_ **= 4.55, *P* < 0.001**	*F*_4,78_ = 0.21, *P* > 0.05	***F***_**4,78**_ **= 2.80, *P* < 0.05**	***F***_**12,78**_ **= 3.35, *P* < 0.001**
**Total Protein content (mg)** (*n* = 3–4)	***F***_**2,73**_ **= 12.11, *P* < 0.001**	***F***_**3,73**_ **= 7.38, *P* < 0.001**	***F***_**2,73**_ **= 6.91, *P* < 0.005**	***F***_**6,73**_ **= 7.69, *P* < 0.001**	*F*_6,73_ = 0.37, *P* > 0.05	*F*_6,73_ = 1.42, *P* > 0.05	***F***_**12,73**_ **= 2.28, *P* < 0.05**

Significant differences (*P* < 0.05) are marked in bold.

**Table 2:** Relationships (Pearson correlation coefficient, *r*) between plant traits of native *Spartina foliosa*, invasive *S. densiflora* and their hybrid *S. densiflora × foliosa* along salinity inundation depth gradients

		**PEPC specific activity**	**IC**_**50**_	**Total protein content**	**Proline concentration**	**Malondialdehyde concentration**
***S. foliosa***	PEPC specific activity	-	*r* = −0.250, *P* = 0.130	*r* = −0.0904, *P* = 0.589	*r* = −0.209, *P* = 0.207	*r* = +0.164, *P* = 0.345
	IC_50_	-	-	*r* = −0.159, *P* = 0.341	*r* = +0.311, *P* = 0.057	*r* = −0.269, *P* = 0.118
	Total protein content	-	-	-	***r* = −0.461, *P* < 0.005**	*r* = +0.286, *P* = 0.096
	Proline concentration	-	-	-	-	*r* = −0.137, *P* = 0.432
	Salinity	*r* = −0.318, *P* = 0.052	***r* = +0.431, *P* < 0.01**	***r* = −0.568, *P* < 0.0001**	***r* = +0.698, *P* < 0.0001**	***r* = −0.462, *P* < 0.005**
	Inundation depth	*r* = −0.271, *P* = 0.099	*r* = +0.130, *P* = 0.435	*r* = +0.279, *P* = 0.090	***r* = +0.356, *P* < 0.05**	*r* = +0.180, *P* = 0.294
***S. densiflora × foliosa***	PEPC specific activity	-	*r* = −0.001, *P* = 0.992	*r* = +0.174, *P* = 0.310	*r* = −0.302, *P* = 0.575	*r* = +0.168, *P* = 0.335
	IC_50_	-	-	*r* = −0.112, *P* = 0.514	*r* = +0.104, *P* = 0.529	*r* = +0.079, *P* = 0.645
	Total protein content	-	-	-	***r* = −0.407, *P* < 0.05**	*r* = +0.071, *P* = 0.690
	Proline concentration	-	-	-	-	***r* = +0.411, *P* < 0.01**
	Salinity	***r* = −0.386, *P* < 0.05**	*r* = +0.173, *P* = 0.291	***r* = −0.387, *P* < 0.05**	***r* = +0.859, *P* < 0.0001**	***r* = +0.340, *P* < 0.05**
	Inundation depth	*r* = +0.035, *P* = 0.841	*r* = +0.038, *P* = 0.819	*r* = +0.241, *P* = 0.157	***r* = +0.340, *P* < 0.05**	*r* = −0.077, *P* = 0.602
***S. densiflora***	PEPC specific activity	-	*r* = −0.053, P = 0.762	*r* = −0.187, P = 0.282	*r* = −0.217, P = 0.211	*r* = +0.133, P = 0.447
	IC_50_	-	-	***r* = +0.365, *P* < 0.05**	***r* = +0.658, *P* < 0.001**	*r* = −0.126, ***P*** = 0.464
	Total protein content	-	-	-	***r* = +0.361, *P* < 0.05**	*r* = −0.012, ***P*** = 0.947
	Proline concentration	-	-	-	-	*r* = −0.342, ***P*** = 0.041
	Salinity	*r* = −0.320, ***P*** = 0.061	***r* = +0.504, *P* < 0.001**	***r* = +0.344, *P* < 0.05**	***r* = +0.798, *P* < 0.0001**	***r* = −0.393, *P* < 0.05**
	Inundation depth	*r* = −0.036, ***P*** = 0.839	*r* = +0.108, ***P*** = 0.525	*r* = +0.231, ***P*** = 0.182	*r* = +0.016, ***P*** = 0.916	*r* = +0.087, ***P*** = 0.614

IC_50_, PEPC phosphorylation state recorded as 50% inhibition of initial PEPC activity by L-malate. *n* = 34–48. Significant results (*P* < 0.05) are marked in bold.

MDA exhibited significant differences among taxa, salinities and inundation depths, and taxa × salinity and salinity × inundation interactions (Table 1). MDA increased with proline concentration and salinity just for the hybrid, whereas both parental species accumulated less MDA at higher salinities. MDA was independent of inundation depth for every taxon (Fig. 1b; Table 2).

### PEPC traits

**Figure 2:**
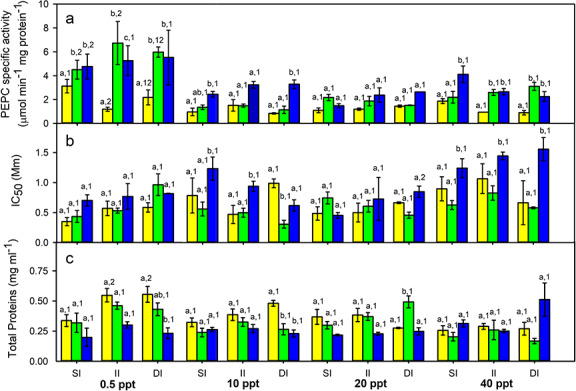
PEPC apparent specific activity (**a**), PEPC phosphorylation state (determined by L-malate assay, IC_50_) (**b**) and total proteins content (**c**) for native *Spartina foliosa* (yellow columns), invasive *S. densiflora* (blue columns) and their hybrid *S. densiflora × foliosa* (green columns) exposed to four salinity treatments (0.5, 10, 20 and 40 ppt) at three inundation depths (shallow inundation (SI), 4.5 cm deep; intermediate inundation (II), 35.5 cm deep; deep inundation (DI), 55.0 cm deep). Values are mean ± SEM (*n* = 3). Different letters indicate significant differences among taxa for the same inundation treatment at every salinity. Different numbers show significant differences among inundation treatments at the same salinity for each taxon (three-way ANOVA, Tukey–HSD, *P* < 0.05)

PEPC apparent specific activity and IC_50_ showed significant differences among taxa, salinities and taxa × salinity, salinity × inundation, and salinity × inundation × taxa interactions (Table 1). *S. foliosa* tended to show lower PEPC apparent specific activity than *S. densiflora*, while their hybrid had intermediate specific activity values. In general, PEPC-specific activity tended to decrease at higher salinities for all taxa, but this relationship was only significant for the hybrid which showed the highest activity values in freshwater. PEPC-specific activity was independent of inundation depth for all taxa, except when under intermediate inundation in freshwater *S. foliosa* expressed its minimum activity and the hybrid its maximum activity values, with the hybrid having higher PEPC-specific activity of either parental species (Fig. 2a; Table 2).

IC_50_ varied among taxa at 10 and 40 ppt salinity, with *S. densiflora* showing the highest values at 10 ppt salinity under lower inundation depths and at hypersalinity under deeper inundation. Thus, IC_50_ increased together with total protein content and proline concentration in *S. densiflora*. IC_50_ values increased with salinity for both parental species, and this relationship was much greater for *S. densiflora* than for *S. foliosa* (Pearson correlation coefficient, *P* < 0.001 and *P* < 0.01, respectively). IC_50_ for the hybrid was independent of salinity. IC_50_ was independent of inundation depth for *S. foliosa* and the hybrid, whereas it increased together with inundation at 20–40 ppt salinity for *S. densiflora* (Pearson correlation coefficient, *P* < 0.05) (Fig. 2b; Table 2).

The total amount of proteins changed significantly among taxa, salinities, and taxa × salinity and salinity × inundation × taxa interactions (Table 1). Total protein content decreased with increasing salinity for *S. foliosa* and its hybrid (especially at hypersalinity), whereas *S. densiflora* accumulated more proteins at higher salinities (Fig. 2c; Table 2).

## Discussion

Native *S. foliosa* showed a moderate sensitivity to salinity in relation to PEPC activity and related functional traits. Thus, *S. foliosa* presented its maximum PEPC amounts in freshwater conditions, reflected in maximum PEPC apparent specific activity combined with minimum activation by phosphorylation (low IC_50_ values). *S. foliosa* tried to compensate for the decrease in PEPC amount at higher salinity levels, with higher activation by phosphorylation as recorded previously for *Spartina maritima x densiflora* (Gallego-Tévar *et al*., 2019a) and mutant *Amaranthus edulis* Speg. (Dever *et al*., 1997). Courtney *et al*. (2016) recorded that PEPC gene transcription was down-regulated at high salinities (>12 ppt), which may explain our recorded decrease in PEPC amounts with increasing salinity. In contrast, the high marsh halophyte *Atriplex halimus* L. increased its foliar PEPC amount at high salinity (c. 30 ppt) (Alla *et al*., 2011). On the other hand, *S. foliosa* PEPC functional traits were independent of inundation depth, except that they had maximum PEPC-specific activity under shallow inundation in freshwater conditions. Thus, *S. foliosa* was moderately sensitive to salinity and to a minor degree, also somewhat sensitive to flooding in relation to its PEPC traits. These results improve the mechanistic understanding of findings from previous studies that characterized *S. foliosa* as moderately tolerant of salinity, given its capacity to colonize new sites by producing viable seeds even under high salinity, and due to its high tolerance to inundation (Gallego-Tévar *et al*., 2020) (see Supplementary information: Literature review). Nevertheless, *S. foliosa* most often occurs in low marsh intertidal zones, which are expected to be highly impacted by SLR (Janousek *et al*., 2019). However, our results suggest that existing natural populations of *S. foliosa* should be able to tolerate a degree of increased salinity and inundation during SLR, as marshes transgress inland where possible and as the new habitat experiences increases in soil salinity and inundation (e.g. Fagherazzi *et al*. 2019). In this scenario, *S. foliosa* has the physiological tolerances to progressively replace upland vegetation as it colonizes the new salt marsh areas. Conservation managers should put highest priority on conservation of undeveloped lands for accommodation space above current high tide lines to facilitate natural migration and colonization of stress-tolerant *S. foliosa*.

Functional responses of PEPC characterized invasive *S. densiflora* as a halophyte highly sensitive to salinity. Maximum PEPC apparent specific activity was recorded in freshwater conditions, and activation by phosphorylation increased markedly at higher salinities as a compensatory mechanism (Gallego-Tévar *et al*., 2019a). The low sensibility to L-malate (low IC_50_ values) indicated a dephosphorylated PEPC at freshwater conditions (Jiao and Chollet, 1992; Wang and Chollet, 1993). Additionally, the increase in activation by phosphorylation with increasing salinity (+84%) co-occurred with high accumulation of free proline (+96%) and total proteins (+23%). Both proline and total protein (including antioxidant enzymes) accumulations are typical responses to salinity stress (Khalid *et al*., 2019), reflecting that the recorded sharp increase in PEPC activation by phosphorylation was also a stress response in *S. densiflora*. Changes in leaf anatomy that have been shown to drive PEPC activity in other *Poaceae* species in response to environmental factors such as light intensity (Ma *et al*., 2017) may also be operating in *Spartina* spp. responses to changes in salinity and flooded conditions. Following previous studies (Castillo *et al*., 2005, Gallego-Tévar *et al*., 2018a, 2020), the invasive halophyte *S. densiflora* was able to tolerate increasing salinity and flooding levels with markedly reduced fitness, but it was able to retain some capacity for seed production. Moreover, IC_50_ increased together with inundation at higher salinities for *S. densiflora*. In this sense, PEPC activity increased twofold after soil flooding in the C-4 plant *Zea mays* L. (Yordanova & Popova, 2007). In view of these results, and given limited resources for invasive species management, the management of *S. densiflora*-invaded marshes should prioritize eradication of new *S. densiflora* populations colonizing more benign (i.e. less saline soils at higher marsh elevations) habitats where growth rates and fruit set will be highest (Nieva *et al*., 2001). In this sense, *S. densiflora* and other *Spartina* taxa are being aggressively managed with the goal of estuary-wide eradication by mechanical and chemical methods in the San Francisco Estuary (Strong and Ayres, 2016). To a lesser degree, *S. densiflora* invasion control is also being carried out in the Humboldt Bay Estuary, northwestern California (Augyte and Pickart, 2014) and at the Odiel Marshes in the Southwest Iberian Peninsula (Castillo and Figueroa, 2009).

Our results on PEPC functional traits are in accordance with the previous characterization of *S. densiflora* as fast-growing species able to tolerate moderate stress levels (Gallego-Tévar *et al*., 2020; see Supplementary information: Literature review). The recorded increase in the phosphorylation state of PEPC at hypersalinity for *S. densiflora* from the San Francisco Estuary contrasted with the opposite response recorded for *S. densiflora* from the Odiel Estuary (Gallego-Tévar *et al*., 2019a). Álvarez *et al*. (2010) reported the existence of local adaptation in an ecotype of *S. densiflora* from high latitudes that was able to compensate low PEPC activation by phosphorylation with increasing amounts of the enzyme at freshwater conditions. Ecotypes with different PEPC activity levels have also been recorded for other wetland plants such as *Phragmites australis* (Cav.) Trin. ex Steud. (Zheng *et al*., 2000). Since *S. densiflora* at measured PEPC levels show higher performance in response to increasing salinity in the San Francisco Estuary than was recorded in the Odiel Estuary, managers should be especially aware of *S. densiflora* invading newly formed wetland restoration areas, and higher salinity salt marshes in recently flooded areas of the San Francisco Estuary.

Finally, Gallego-Tévar *et al*. (2020) described the hybrid *S. densiflora* × *foliosa* as a taxon relatively tolerant of both salinity and inundation. This is in accordance with our results in relation to its PEPC responses of the taxon, in which activity was mostly independent of both stress factors. However, in our experiment, the hybrid did express its maximum PEPC apparent specific activity in freshwater conditions. The sensitivity of the hybrid to salinity was reflected in oxidative stress, showing increasing MDA levels (+20%) at higher salinities. However, the hybrid tended to show mostly intermediate responses compared to responses of both parental species (additive inheritance). The hybrid had higher PEPC specific activity than *S. foliosa* (+70%) and *S. densiflora* (+15%) (*best-parent heterosis*) in freshwater under intermediate inundation depth. This heterotic response in PEPC enzymatic level coincided with the hybrid’s maximum net photosynthesis rates and maximum vegetative fitness (Gallego-Tévar *et al*., 2019c). Measured PEPC responses support the high stress tolerance of the *S. densiflora* × *foliosa* hybrid to increasing salinity, inundation and their interaction. Therefore, eradication of the hybrid should be the highest management priority. The hybrid should be eradicated before it potentially evolves to become a fertile, highly competitive and wide-spread allopolyploid species that will reduce local biodiversity, as has previously demonstrated in the case of the evolution of *Spartina anglica* C.E.Hubb. in European salt marshes (Aïnouche *et al*. 2004). In line with our suggestions on management of *Spartina* hybrid, the invasions by different hybrids between *S. foliosa*, *S. alterniflora* and *S. densiflora* are already under management as invasive species in the San Francisco Bay (Kerr *et al*., 2016). Our study shows that the responses of key functional physiological traits to increasing environmental stresses, such as specific enzymatic activities like PEPC in C4 species, interpreted together with other functional trait responses, serve as biological indicators that provide a mechanistic framework for improved ecosystem management practices in a scenario of biological invasions and climate change.

## Supplementary Material

Supplementary_Fig_1_coaa053

Supplementary_Literature_Review_coaa053
