# Supplementary material for: From physiology to salt marsh management challenges with sea level rise: the case of native Spartina foliosa, invasive S. densiflora and their hybrid
Source: Conserv Physiol. 2020 Jul 1;8(1):coaa053. doi: 10.1093/conphys/coaa053 (PMC7327128; doi:10.1093/conphys/coaa053)
Supplement: Supplementary_Fig_1_coaa053 [file supplementary_fig_1_coaa053.docx]

Gallego-Tévar_et_al_Conservation_Physiology_Supplementary Information - Figures

**From physiology to salt marsh management challenges with sea level rise: the case of native *Spartina foliosa*, invasive *S. densiflora* and their hybrid**

Blanca Gallego-Tévar, Procopio Peinado-Torrubia, Rosario Álvarez, Brenda J. Grewell and Jesús M. Castillo

**Supplementary Information: Figure S1**


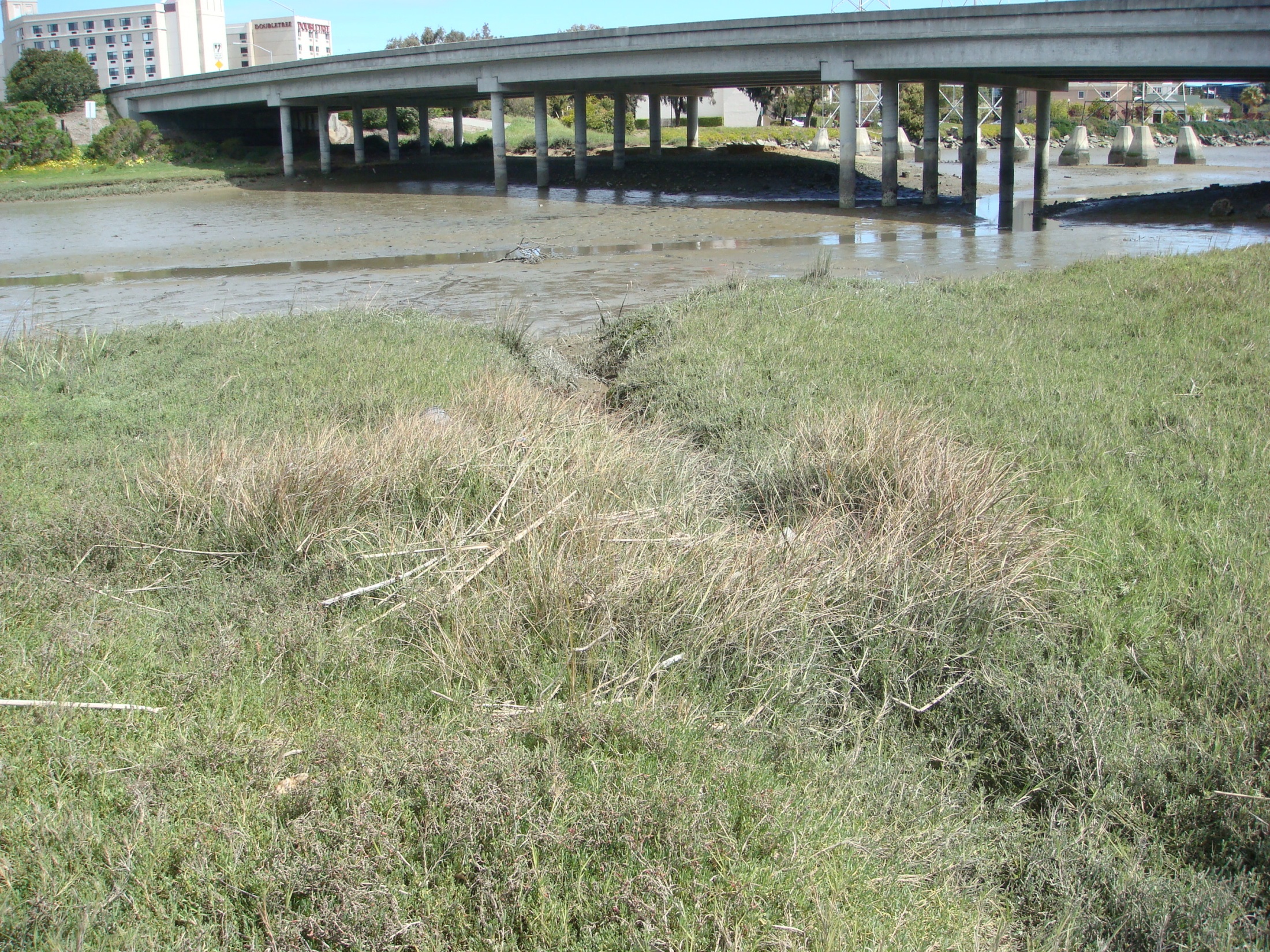

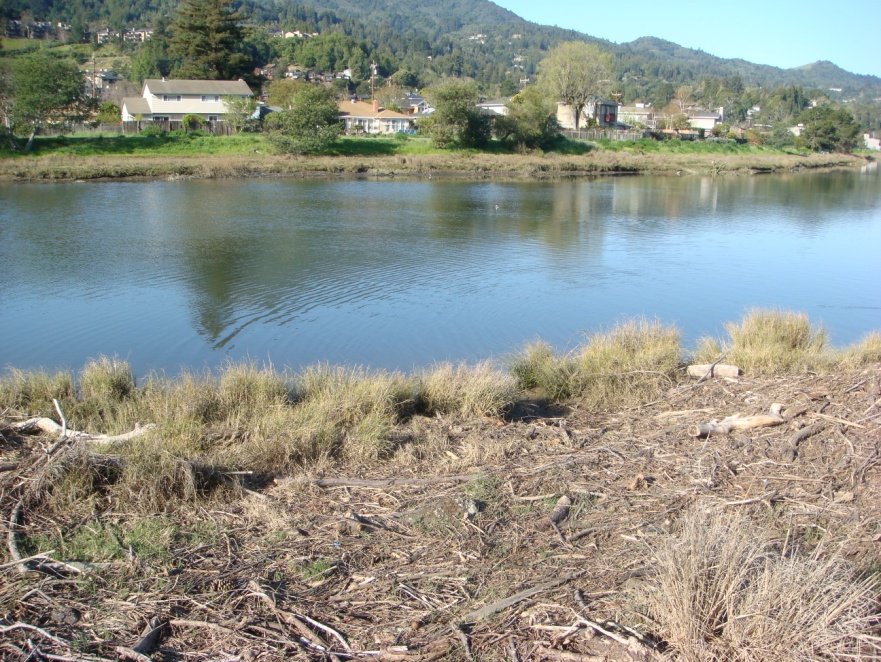

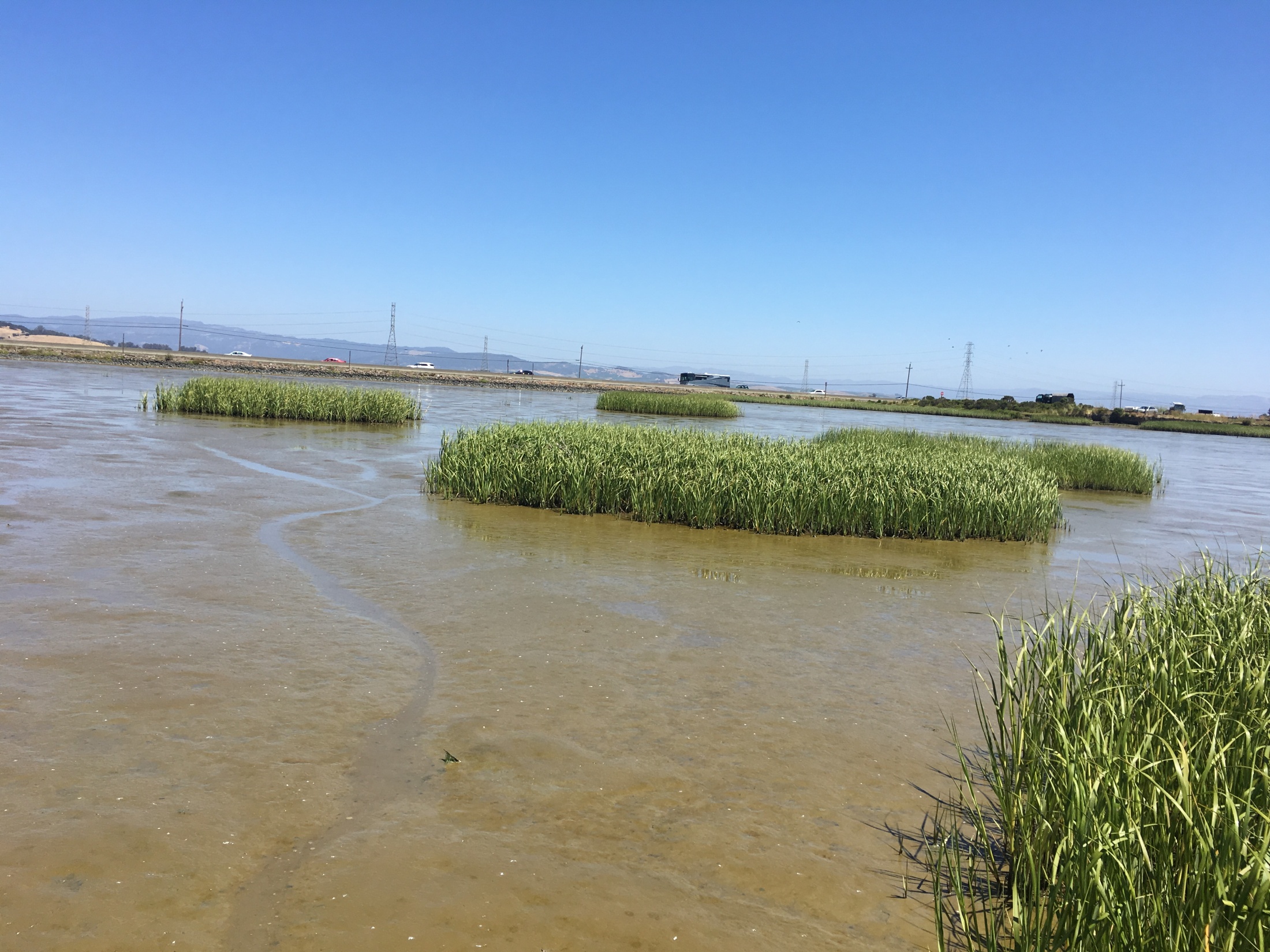


**a**

**b**

**cA**

**Supplementary Figure S1:** Native *Spartina foliosa* colonizing a low marsh (a), invasive *S. densiflora* (b) and the hybrid *S. densiflora x foliosa* (c) growing in middle marshes in the San Francisco Estuary (California, USA)
