# Supplementary material for: From physiology to salt marsh management challenges with sea level rise: the case of native Spartina foliosa, invasive S. densiflora and their hybrid
Source: Conserv Physiol. 2020 Jul 1;8(1):coaa053. doi: 10.1093/conphys/coaa053 (PMC7327128; doi:10.1093/conphys/coaa053)
Supplement: Supplementary_Literature_Review_coaa053 [file supplementary_literature_review_coaa053.docx]

Gallego-Tévar_et_al_Conservation_Physiology_Supplementary Information -pdf file

**From physiology to salt marsh management challenges with sea level rise: the case of native *Spartina foliosa*, invasive *S. densiflora* and their hybrid**

Blanca Gallego-Tévar, Procopio Peinado-Torrubia, Rosario Álvarez, Brenda J. Grewell and Jesús M. Castillo

**Supplementary Information: Literature review**

***Responses of three focal* Spartina *spp. to flooding and/or salinity stress***

We systematically searched from 1900 to the present using all bibliographic databases included in the Web of Science for studies on anatomical, morphological, ecophysiological, growth and sexual reproduction responses of the three studied *Spartina* taxa to salinity and flooding stress. We used as topics the search terms “*Spartina foliosa*”, “*Spartina densiflora*”, “*Spartina densiflora x foliosa*”, “salinity”, “inundation”, and words starting with “salt”, and “flood”. The initial research identified 211 papers that were searched and reviewed to select 36 and 27 references related to salinity and inundation, respectively. We reviewed and tabulated a summary of the data on *Spartina* responses to salinity and flooding from all of these papers (Supplementary Tables S1 – S3).

Both *Spartina* parental species and their hybrid responded to increasing salinity mainly by producing thicker and rolled leaves and short shoots, accumulating organic solutes and reducing their chlorophyll concentration and their foliar carbon:nitrogen ratio (C:N). In addition, *S. foliosa* and the hybrid increased energy dissipation from the photosynthetic apparatus at hypersalinity. Moreover, the hybrid showed high rhizomes reserves and transgressive growth at high salinities, whereas *S. densiflora* lowered its rhizome reserves, increased its root biomass and reduced its net photosynthesis rate due to stomatal closure and photochemical stress (Supplementary Tables 1 and 2).

In response to increasing inundation, the three studied taxa produced more roots, and thin and rolled leaves with high salt excretion rates and high concentrations of organic solutes. Moreover, both parental species produced short shoots and accumulated low C storage reserves with more inundation. Additionally, with increasing inundation, *S. foliosa* increased production of rhizome aerenchyma, had constant net photosynthesis rates and medium growth rates. *S. foliosa* also maintained above-ground biomass accumulation and sexual reproduction even under high inundation. In contrast, with increasing inundation *S. densiflora* increased root aerenchyma, shortened its leaves, produced leaves with lower total N, chlorophyll pigments and stomatal density. With increased inundation stress, *S. densiflora* also increased photosynthetic thermal dissipation and anti-oxidant enzymes activity, and had lower net photosynthetic rates resulting in reduced overall growth, sexual reproductive output and establishment by seeds. Previous studies also show *S. densiflora* formed biannual (not perennial) populations under high flooding conditions. Under increasing inundation, the hybrid decreased allocation to rhizome biomass, had higher photosynthetic pigment concentrations, and constant net photosynthetic rates (Supplementary Tables 1 and 3).

In response to the combined effects of salinity and inundation depth, the three studied *Spartina* taxa had increased foliar N concentrations, increased salt excretion rates, and they accumulated glycinebetaine at increasing inundation except at hypersalinity (Supplementary Table 1).

In view of this literature review, *S. foliosa* showed few stress responses due to low to moderate salinity and inundation sensitivity. In contrast, *S. densiflora* showed high phenotypic plasticity reflecting high salinity and inundation sensitivity, whereas the hybrid showed both high salinity and inundation tolerance.

**Supplementary Table 1:** Main anatomical, morphological, physiological, growth and sexual reproductive responses to salinity and inundation stress and their combination for native *Spartina foliosa*, invasive *S. densiflora* and their hybrid *S. densiflora x foliosa* based on literature review.

| **Taxon** | **Increasing salinity stress** | **Increasing inundation stress** | **Increasing salinity and inundation stresses** |
| --- | --- | --- | --- |
| ***S. foliosa*** | - Reduced shoot height.  - Thick and rolled leaves with lower leaf C:N ratio.  - Accumulation of organic solutes.  -Reduced chlorophyll concentration.  - Increased photosynthetic thermal dissipation at hypersalinity. | - More roots and more rhizomes with less reserves and more aerenchyma.  - Reduced shoot height.  - Thin and rolled leaves with high salt excretion rate and high organic solutes concentration.  - Constant net photosynthesis rate.  - Mid growth, above-ground biomass and sexual reproduction. | - High leaf Na concentration, salt excretion rate and leaf nitrogen concentration.  - High leaf glycinebetaine accumulation with inundation, but not at hypersalinity. |
| ***S. densiflora*** | - Less root biomass accumulation and reduced rhizome reserves.  - Reduced shoot height.  - Short, thick and rolled leaves with lower leaf C:N ratio and accumulation of organic solutes.  - Reduced/increased chlorophyll concentration.  - Low net photosynthesis rate due to stomatal enclosure and photochemical stress.  - Low growth and biomass accumulation. | - More roots with more aerenchyma and less rhizome reserves.  - Reduced shoot height.  - Short, thin and rolled leaves with low nitrogen concentration and stomata density, high salt excretion, glycinebetaine accumulation and anti-oxidant enzymes activity.  - Reduced chlorophyll concentration.  - Increased photosynthetic thermal dissipation and low net photosynthesis rate.  - Reduced growth, sexual reproduction and establishment  - Biannual populations | - High salt excretion rate and leaf nitrogen concentration.  - High leaf glycinebetaine accumulation with inundation, but not at hypersalinity. |
| ***S. densiflora x foliosa*** | - High rhizome reserves.  - Reduced shoot height.  - Thick and rolled leaves.  - Accumulation of organic solutes.  - Reduced chlorophyll concentration.  - Increased photosynthetic thermal dissipation at hypersalinity.  - Transgressive growth. | - More roots and fewer rhizomes.  - Thin and rolled leaves with high salt excretion rate and glycinebetaine concentration.  - Increased photosynthetic pigments concentration.  - Constant net photosynthesis rate. | - High salt excretion rate and leaf nitrogen concentration.  - High leaf glycinebetaine accumulation with inundation, but not at hypersalinity. |

Reference sources: *S. foliosa*: Brand *et al*., 2012; Cain and Harvey 1983; Cohen *et al*., 2014; Gallego-Tévar *et al*., 2019b; Janousek *et al*., 2016; Kuramoto and Brest, 1979; Lee *et al*., 2016; Pearcy and Ustin, 1984; Phleger, 1971; Smart and Barko, 1978; Trnka & Zedler, 2000; Ustin *et al*., 1982; Watson and Byrne, 2009; Wrad *et al*., 2003; Zedler *et al*., 1999; *S. densiflora*: Abbas *et al*., 2012; Calado *et al.,* 2015; Canalejo *et al*., 2014; Cantero *et al*., 1998; Castillo *et al*., 2000, 2005, 2008ª, b, 2009, 2014, 2016; Castillo and Figueroa, 2009b; Di Bella *et al*., 2014; Gallego-Tévar *et al*., 2018a, b; Gallego-Tévar *et al*., 2019b; González-Trilla *et al*., 2010; Grewell *et al*., 2016; Idaszkin *et al*., 2014; Infante-Izquierdo *et al*., 2019; Kittelson and Boyd, 1997; Maricle *et al*., 2006; Maricle *et al*., 2007; Mateos-Naranjo and Redondo-Gómez, 2016; Mateos-Naranjo *et al*., 2007, 2008, 2010, 2013; Mirlean and Costa, 2017; Montemayor *et al*., 2014; Nieva *et al*., 1999, 2001, 2003; Pascual *et al*., 2015; Redondo-Gómez *et al*., 2011; Vicari *et al*., 2002; *S. densiflora x foliosa*: Gallego-Tévar *et al*., 2019b, c; Lee *et al*., 2016.

**Supplementary Table 2:** Biochemical, anatomical, morphological and biomass allocation, gas exchange, growth and sexual reproduction responses to increasing salinity for Californian native *Spartina foliosa* (*Sf*), invasive *S. densiflora* (*Sd*) and their hybrid S*. densiflora x foliosa* (*Sdxf*). Superscripts indicate references (see below).

| **Taxon** | **Biochemical traits** | **Anatomical traits** | **Morphological and allocation traits** | **Gas exchange** | **Growth and sexual reproduction** |
| --- | --- | --- | --- | --- | --- |
| ***S. foliosa*** | *Foliar C:N↓^9^  *[Chl]↓> 15 ppt; Chl:Car↓^2^ Chl*a*:Chl*b*↑ at 40 ppt^9^  *[Proline↑at 40 ppt^9^  *[Foliar K]↑^27^  *[Glycineb*et*aine]↑^9^  *Salt excr*et*ion↑> 20 ppt^9^ | * [Rhizome TNC]↕^9^ | *Shoot length↓^9,11,28^  *Leaf rolling↑^9^  *SLA ↓^9^ | *Pn*et* , Gs↓ at 40 ppt^9,11^  *Max. Gs at 10 ppt^9,11^ | *Biomass↕^9^  *Growth and sexual reproduction↓ at 40 ppt^9,10,11,26^  *Max. growth in freshwater^28^ |
| ***S. densiflora*** | *Foliar C:N↓^4,8,9,13^  *[Chl] ↓> 15 ppt^4,8,9,20^; Chl:Car↓ Chl*a*:Chl*b*↑at 40 ppt^8.9^; [Chl], [Car] ↑^13,25^  * LWC^8^↕; LWC↓^13^  *[Proline]↑> 15 ppt^4,8,9^  *[Glycineb*et*aine]↑^4,9^  *Salt excr*et*ion↑> 20 ppt^8,9^  *Max. APX activity at 10 ppt^12^ | *[Rhizome TNC]↑ at c. 10 ppt^2^ and↓ at 40 ppt^9^  *Stomata density↓ at c. 10 ppt^7^ | *LAI↑ at c. 10-15 ppt^2,6^  *RMR↑^2,9^  *Leaf size↓^22^  *Leaf rolling↑^4,9^  *Max. seedling height at c. 10 ppt^17^  *Shoot length↓^9,12,17,20,25^  *SLA↓^9,13^ | *Max. Pn*et* at 10 ppt^12^  *Pn*et* , Gs ,фPSII and Fv/Fm↓ > 15-20 ppt^1,8,5,9,12,13,14,15,25^; Pn*et* , Gs ,фPSII and Fv/Fm↕^19^  *NPQ↑ > 20 ppt^8,25^  *Fv/Fm↓ at 0.5 ppt^1,13^  * WUE↕^8,13^; WUE^15,25^ | *Growth and sexual reproduction↓ > 20 ppt^1,9,10,12,13,17,19,20,25^  *Max. growth at c. 10 ppt^8,9,12,13^  *Max. AGB and BGB at c. 10-15 ppt^2,6,12,13,19^  *AGB and BGB↓ > 15 ppt^6,12,13,22,24,25^ |
| ***S. densiflora x foliosa*** | *Foliar C:N↕^9^  *[Chl]↓> 15 ppt; Chl:Car↓ Chl*a*:Chl*b*↑at 40 ppt^9^  *[Proline]↑at 40 ppt^9^  *[Glycineb*et*aine]↑^9^  *Salt excr*et*ion↑> 20 ppt^9^ | *[Rhizome TNC]↑^9^ | *Shoot length↓^9^  *Leaf rolling↑^9^  *SLA↓^9^ | *Pn*et* , Gs↓ at 40 ppt^9^  *Max. Gs at 10 ppt^9^ | *Growth and sexual reproduction↓ at 40 ppt^9,10^  *Growth: *Sdxf* > *Sd* > *Sf*^10^ |

↕ Constant values; ↑ Higher values; ↓ Lower values with increasing salinity.

Abbreviations: APX, ascorbate peroxidase; C, carbon; Car, Carotenoids; Chl, chlorophyll; Fv/Fm, maximum quantum yield of Photosystem II photochemistry; Gs, stomatal conductance; K, potassium; LAI, leaf area index; LWC, leaf water content; N, nitrogen; Pn*et*, n*et* photosynthesis rate; RMR, root mass ratio; SLA, specific leaf area; TNC, non-structural carbohydrates; WUE, water use efficiency; фPSII, quantum yield of Photosystem II photochemistry

**Supplementary Table 3:** Biochemical, anatomical, morphological and biomass allocation, gas exchange, growth and sexual reproduction responses to increasing inundation for Californian native *Spartina foliosa* (*Sf*), invasive *S. densiflora* (*Sd*) and their hybrid S*. densiflora x foliosa* (*Sdxf*). Superscripts indicate references (see below).

| **Taxon** | **Biochemical traits** | **Anatomical traits** | **Morphological and allocation traits** | **Gas exchange** | **Growth and sexual reproduction** |
| --- | --- | --- | --- | --- | --- |
| ***S. foliosa*** | *Leaf C:N↓^12^  *Chl*a*:Chl*b* ↓^12^  *Salt excr*et*ion↑, *Sf* > *Sd*^12^  *[Proline]↑^12^  *[Glycineb*et*aine]↑^12^ | *Rhizome porosity: *Sf* > *Sd*^12^  *↕ Root porosity^12^  *[Rhizome TNC]↓^12^ | *RMR↑^12^  *Shoot height↓^12^  *Leaf rolling↑^12^  *SLA↑^12^  *AGB and shoot density↓ with increasing elevation^20^ | * Pn*et* ↕ ; Pn*et* and Gs: *Sf* = *Sdxf* > *Sd*^12^ | *Growth and biomass↓^12^  *Sexual reproduction↓^12^ |
| ***S. densiflora*** | *[Foliar N] ↓ on saturated soil^1^  *Leaf C:N ↓^12^  *[Chl] ↓ > 2 cm water deep^1^  *Chl:Car↓at low Eh^7^  *Salt excr*et*ion↑^12^  *[Glycineb*et*aine]↑^12^  *Cytochrome *c* oxidase and ADH↑^14^ | *Aerenchyma in roots↑^3,12^  *[Rhizome TNC] ↓ at low Eh^8^ and high inundation depth^12^  *Stomata density↓ c. 80% WC^11^ | *Shoot height and density↓ at low Eh^8^ & elevation^5,11,12,19^  *Shoot density↑ at low elevation and low Eh^11^ in Odiel; ↕Shoot density with inundation in Argentina^14^  *RMR↑^12^  *Leaf rolling↑ at low Eh^7^ and deep inundation^12^  *Leaf length↓ at low Eh^7^  *SLA↑^12^ | *WUE↓ > 4 cm water deep^1^  *Pn*et* and Fv/Fm↓ at low elevations^3^ and Eh^10^ and permanent flooding^17^ | *Establishment and sexual reproduction↓ > 2 cm water deep^1,12^  *Growth and AGB↓ > 4 cm water deep^1^ and low elevations^3,5,11,12,15,16,17,18,19^  *Biannual populations at low elevations^2^ |
| ***S. densiflora x foliosa*** | *Leaf C:N ↓^12^  *Salt excr*et*ion↑ ^12^  *[Glycineb*et*aine]↑^12^  *[Chl] and [Car]↑ ^12^ | *Root porosity↕ ^12^  *[Rhizome TNC]↕ ^12^ | *Shoot height↓. *Sdxf* > *Sf* = *Sd*^12^  *Leaf rolling↑^12^  *SLA↑^12^  *Roots↑ and rhizomes ↓^12^ | *Pn*et*↕^12^ | *Growth and biomass↓^12^  *Sexual reproduction↓^12^ |

↕ Constant values; ↑ Higher values; ↓ Lower values with increasing inundation.

Abbreviations: ADH, alcohol dehydrogenase; AGB, above-ground biomass; C, carbon; Car, Carotenoids; Chl, chlorophyll; Eh, sediment redox potential; Fv/Fm, maximum quantum yield of Photosystem II photochemistry; Gs, stomatal conductance; N, nitrogen; Pn*et*, n*et* photosynthesis rate; SLA, specific leaf area; RMR, root mass ratio; TNC, non-structural carbohydrates; WUE, water use efficiency; фPSII, quantum yield of Photosystem II photochemistry.

**References**

**Supplementary Table 2**

^1^Castillo *et al*., 2005; ^2^Grewell *et al*., 2016; ^3^Gallego-Tévar *et al*., 2018b; ^4^Castillo *et al*., 2014; ^5^Nieva *et al*., 2003; ^6^Castillo *et al*., 2016; ^7^Infante-Izquierdo *et al*., 2019; ^8^Gallego-Tévar *et al*., 2018a; ^9^Gallego-Tévar *et al*., 2019; ^10^Lee *et al*., 2016; ^11^Pearcy and Ustin, 1984; ^12^Redondo-Gómez *et al*., 2011; ^13^Mateos-Naranjo *et al*., 2010; ^14^Maricle *et al*., 2007; ^15^Nieva *et al*., 1999; ^16^Cantero *et al*., 1998; ^17^Kittelson and Boyd, 1997; ^18^Mirlean and Costa, 2017; ^19^Mateos-Naranjo and Redondo-Gómez, 2016; ^20^Nieva *et al*., 2001; ^21^Mateos-Naranjo *et al*., 2008; ^22^Pascual *et al*., 2015; ^23^Canalejo *et al*., 2014; ^24^Di Bella *et al*., 2014; ^25^Mateos-Naranjo *et al*., 2013; ^26^, Kuramoto and Brest, 1979; ^27^Smart and Barko, 1978; ^28^Phleger, 1971

**Supplementary Table 3**

^1^Abbas *et al*., 2012; ^2^Castillo and Figueroa, 2009; ^3^Castillo *et al*., 2000; ^5^Castillo *et al*., 2008; ^6^Castillo *et al*., 2009; ^7^Castillo *et al*., 2014; ^8^Castillo *et al*., 2016; ^9^Gallego-Tévar *et al*., 2018a; ^10^Nieva *et al*., 2003; ^11^Infante-Izquierdo *et al*., 2019; ^12^Gallego-Tévar *et al*., 2019; ^13^Maricle *et al*., 2006; ^14^Idaszkin *et al*., 2014; ^15^Vicari *et al*., 2002; ^16^Montemayor *et al*., 2014; ^17^Mateos-Naranjo *et al*., 2007; ^18^Calado *et al.,* 2015; ^19^González-Trilla *et al*., 2010; ^20^Janousek *et al*., 2016

Abbas AM, Rubio‐Casal AE, De Cires A, Figueroa ME, Lambert AM,Castillo JM (2012) Effects of flooding on germination and establishment of the invasive cordgrass *Spartina densiflora*. *Weed Res*52: 269-276.

Calado ML, Carvalho L, Pang KL, Barata M (2015) Diversity and ecological characterization of sporulating higher filamentous marine fungi associated with *Spartina maritima* (Curtis) Fernald in two Portuguese salt marshes. *Microb Ecol* 70: 612-633.

Canalejo A, Martínez-Domínguez D, Córdoba F, Torronteras R (2014) Salt tolerance is related to a specific antioxidant response in the halophyte cordgrass, *Spartina densiflora. Estuar Coast Shelf Sci* 146: 68–75.

Cantero JJ, Cisneros JM, Zobel M, Cantero A (1998) Environmental relationships of vegetation patterns in saltmarshes of central Argentina. *Folia Geobot* 33: 133.

Castillo JM, Fernández‐Baco L, Castellanos EM, Luque CJ, Figueroa ME, Davy AJ (2000) Lower limits of *Spartina densiflora* and *S. maritima* in a Mediterranean salt marsh determined by different ecophysiological tolerances. *J Ecol* 88: 801-812.

Castillo JM, Rubio-Casal AE, Redondo S, Álvarez-López AA, Luque T, Luque C, Nieva FJ, Castellanos EM, Figueroa EM (2005) Short-term responses to salinity of an invasive cordgrass. *Biol Invasions* 7: 29–35.

Castillo JM, Mateos-Naranjo E, Nieva FJ, Figueroa E (2008) Plant zonation at salt marshes of the endangered cordgrass *Spartina maritima* invaded by *Spartina densiflora*. *Hydrobiologia* 614: 363.

Castillo JM, Figueroa E (2009) Effects of abiotic factors on the life span of the invasive cordgrass *Spartina densiflora* and the native *Spartina maritima* at low salt marshes. *Aquat Ecol* 43: 51-60.

Castillo JM, Ayres DR, Leira-Doce P, Bailey J, Blum M, Strong DR, Luque T, Figueroa E (2010) The production of hybrids with high ecological amplitude between exotic *Spartina densiflora* and native *S. maritima* in the Iberian Peninsula. *Divers Distrib* 16: 547–558.

Castillo JM, Brewell JG, Pickart A, Bortolus A, Peña C, Figueroa E, Sytma M (2014) Phenotypic plasticity of invasive *Spartina densiflora* (Poaceae) along a broad latitudinal gradient on the Pacific Coast of North America. *Am J Bot* 101: 448–458.

Castillo JM, Grewell BJ, Pickart AJ, Figueroa ME, Sytsma M. 2016. Variation in tussock architecture of the invasive cordgrass *Spartina densiflora* along the Pacific Coast of North America. *Biol Invasions* 18: 2159–2174.

Castillo JM, Gallego-Tévar B, Figueroa E, Grewell JG, Vallet D, Rousseau H, Keller J, Lima O, Dréano S, Salmon A, Aïnouche M *(*2018). Low genetic diversity contrasts with high phenotypic variability in heptaploid *Spartina densiflora* populations invading the Pacific coast of North America. *Ecol Evol* 8: 4992–5007.

Di Bella CE, Jacobo E, Golluscio RA, Rodríguez AM (2014) Effect of cattle grazing on soil salinity and vegetation composition along an elevation gradient in a temperate coastal salt marsh of Samborombón Bay (Argentina). *Wetlands Ecol Manage* 22: 1-13.

Gallego-Tévar B, Rubio-Casal AE, de Cires A, Figueroa E, Grewell BJ, Castillo JM (2018a) Phenotypic plasticity of polyploid plant species promotes transgressive behaviour in their hybrids. *AoB Plants* 10: ply055.

Gallego-Tévar B, Curado G, Grewell BJ, Figueroa ME, Castillo JM. (2018b) Realized niche and spatial pattern of native and exotic halophyte hybrids. *Oecologia* 188: 849-862.

Gallego-Tévar B, Grewell B. J, Futrell C.J, Drenovsky R. E, Castillo, JM (2019) Interactive effects of salinity and inundation on native *Spartina foliosa*, invasive *S.* *densiflora* and their hybrid from San Francisco Estuary, California. *Ann Bot* mcz170.

Grewell BJ, Castillo JM, Skaer Thomason MJ, Drenovsky RE (2016) Phenotypic plasticity and population differentiation in response to salinity in the invasive cordgrass *Spartina densiflora*. *Biol Invasions* 18: 2175–2187.

Idaszkin YL, Bortolus A, Bouza PJ (2014) Flooding effect on the distribution of native austral cordgrass *Spartina densiflora* in Patagonian salt marshes. *J Coast Res* 30: 59 – 62.

Infante-Izquierdo MD, Gallego-Tévar B, Sánchez-Gullón E, Nieva FJJ, Grewell BJ, Castillo JM, Muñoz-Rodríguez AF (2019) Morphological and anatomical evidence supports differentiation of new interspecific hybrids from native *Spartina maritima* and invasive *S. densiflora* (Poaceae, subfamily Chloridoideae). *Pl Syst Evol* 305: 531-547.

Kittelson P M, Boyd MJ (1997) Mechanisms of expansion for an introduced species of cordgrass, *Spartina densiflora*, in Humboldt Bay, California. *Estuaries* 20: 770-778.

Kuramoto RT, Brest DE (1979) Physiological response to salinity by four salt marsh plants.*Bot Gaz*140: 295-298.

Lee AK, Ayres DR, Strong DR (2016) Responses to salinity of *Spartina* hybrids formed in San Francisco Bay, California (*S. alterniflora x foliosa* and *S. densiflora x foliosa*). *Biol Invasions* 18: 2207–2219.

Maricle BR, Crosier JJ, Bussiere BC, Lee RW (2006) Respiratory enzyme activities correlate with anoxia tolerance in salt marsh grasses. *J Exp Mar Biol Ecol*337: 30-37.

Maricle BR, Lee RW, Hellquist CE, Kiirats O, Edwards GE (2007) Effects of salinity on chlorophyll fluorescence and CO 2 fixation in C4 estuarine grasses. *Photosynthetica* 45: 433-440.

Mateos-Naranjo E, Redondo-Gómez S, Silva J, Santos R, ME Figueroa (2007) Effect of prolonged flooding on the invader *Spartina densiflora* Brong. *J Aquat Plant Manag* 45: 121–123.

Mateos-Naranjo E, Redondo-Gómez S, Luque CJ, Castellanos EM, Davy AJ, Figueroa ME (2008) Environmental limitations on recruitment from seed in invasive *Spartina densiflora* on a southern European salt marsh. *Estuar Coast Shelf Sci* 79: 727-732.

Mateos-Naranjo E, Redondo-Gómez S, Figueroa ME (2010) Synergic effect of salinity and CO_2_ enrichment on growth and photosynthetic responses of the invasive cordgrass *Spartina densiflora*. *J Exp Bot* 61: 1643–1654.

Mateos-Naranjo E, Andrades-Moreno L, Davy AJ (2013) Silicon alleviates deleterious effects of high salinity on the halophytic grass *Spartina densiflora*. *Plant Physiol Biochem* 63: 115-121.

Mateos-Naranjo E, Redondo-Gómez S (2016) Interpopulation differences in salinity tolerance of the invasive cordgrass *Spartina densiflora*: implications for invasion process. *Estuar Coast* 39: 98-107.

Mirlean N, Costa CS (2017) Geochemical factors promoting die-back gap formation in colonizing patches of *Spartina densiflora* in an irregularly flooded marsh. *Estuar Coast Shelf Sci* 189: 104-114.

Montemayor DI, Canepuccia AD, Pascual J, Iribarne OO (2014) Aboveground biomass patterns of dominant *Spartina* species and their relationship with selected abiotic variables in Argentinean SW Atlantic marshes. *Estuar Coast* 37: 411-420.

Nieva FJJ, Castellanos EM, Figueroa ME, Gil F (1999) Gas exchange and chlorophyll fluorescence of C3 and C4 saltmarsh species. *Photosynthetica* 36: 397-406.

Nieva FJJ, Díaz-Espejo A, Castellanos EM, Figueroa ME (2001) Field variability of invading populations of *Spartina densiflora* Brong. in different habitats of the Odiel Marshes (SW Spain). *Estuar Coast Shelf Sci* 52: 515–527.

Nieva FJJ, Castillo JM, Luque CJ, Figueroa ME (2003) Ecophysiology of tidal and non-tidal populations of the invading cordgrass *Spartina densiflora*: seasonal and diurnal patterns in a Mediterranean climate*. Estuar Coast Shelf Sci* 57: 919-928.

Pascual J, Canepuccia AD, Alberti J, Daleo P, Iribarne O (2015) Rainfall intensity modulates the interaction between the marsh cordgrass *Spartina densiflora* and the mouse Akodon azarae. *Mar Ecol Prog Ser*523: 71-80.

Pearcy RW, Ustin SL (1984) Effects of salinity on growth and photosynthesis of three California tidal marsh species. *Oecologia* 62: 68-73.

Phleger CF (1971) Effect of salinity on growth of a salt marsh grass. *Ecology* 52: 908-911.

Redondo-Gómez S, Andrades-Moreno L, Mateos-Naranjo E, Parra R, Valera-Burgos J, Aroca R (2011) Synergic effect of salinity and zinc stress on growth and photosynthetic responses of the cordgrass, *Spartina densiflora*. *J Exp Bot* 62: 5521-5530.

Smart RM, Barko JW (1978) Influence of sediment salinity and nutrients on the physiological ecology of selected salt marsh plants. *Estuar Coast Mar Sci* 7: 487-495.

Trilla GG, De Marco S, Marcovecchio J, Vicari R, Kandus P (2010) Net primary productivity of *Spartina densiflora* Brong in an SW Atlantic Coastal salt marsh. *Estuar Coast* 33: 953-962.

Trnka S, Zedler JB (2000) Site conditions, not parental phenotype, determine the height of *Spartina foliosa*. *Estuaries* 23: 572-582.

Vicari RL, Fischer S, Madanes N, Bonaventura SM, Pancotto V (2002) Tiller population dynamics and production on *Spartina densiflora* (Brong) on the floodplain of the Parana River, Argentina. *Wetlands* 22: 347-354.
